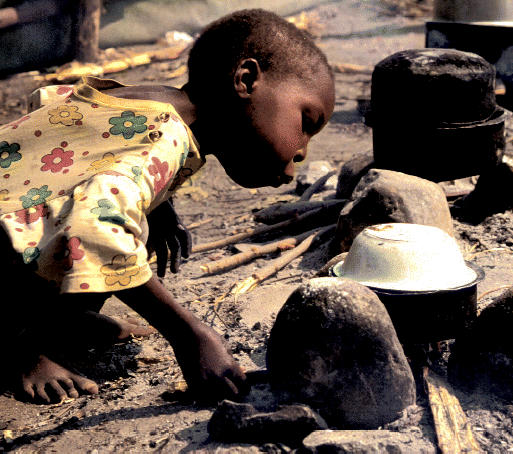# The Beat

**Published:** 2005-09

**Authors:** Erin E. Dooley

## Watching Mines in Eastern Europe

In January 2000, cyanide from a Romanian gold mine spilled into the Tisza River, killing nearly all the aquatic life and fouling the drinking water of millions of people. To help avoid such incidents in the future, government officials from a dozen southeastern European countries came together in May 2005 in Cluj-Napoca, Romania, and signed on to a new strategy calling for detailed site assessments for mines of concern, higher health and environmental standards for new mines, and plans for their eventual closure. The agreement also calls for early warning systems to warn countries downstream of mining-related pollution incidents. More than 150 mining operations exist in the area; more than a third have been labeled by the UN Environment Programme as posing a serious risk to human health, the environment, and regional stability.

## Smoking Ends Up on Cutting-Room Floor

“Bollywood,” the Indian film industry and the world’s largest producer of films, has been ordered by the Indian government to cut movie and TV scenes showing actors smoking, effective October 2005. Announcement of the ban set off a flurry of dissent from Indian film makers, who see it as censorship, although some actors have expressed support for the decision. Health minister Anbumani Ramadoss said the ban could save millions of children who would otherwise start smoking “under the influence of movies.” This new law, which also requires listing of tar and nicotine content on cigarette packaging, comes just a year after India banned smoking in public places and forbade tobacco firm advertising in and sponsorship of sporting events. Each year more than 800,000 Indians die smoking-related deaths.

## The Power of Pachyderm Poo

The Rosamund Gifford Zoo of Syracuse, New York, is investigating a potential new source of renewable energy, one that is based on the daily output of the zoo’s own residents, especially its six elephants—the zoo is looking at the half-ton of elephant manure produced each day as a feed-stock to produce methane or hydrogen for a fuel cell or generator. The zoo is also studying whether it could use the manure from a number of its other large animals. Using the animal waste would not only provide fuel, but also save the zoo money in disposal fees as well as the fossil fuels used to transport the waste. Many U.S. farms already use animal waste for power production.

## Herbal Answers for Deadly Diseases

Ohio State University researchers have found that extracts from two Mojave Desert plants can kill the parasites that cause leishmaniasis and African sleeping sickness. These diseases afflict millions, primarily in developing nations, and are usually fatal if left untreated. Drugs based on chemicals from the dotted dalea and the Mojave dalea may offer a cheaper, safer, and more expedient alternative to the costly and sometimes nephrotoxic drugs currently used to treat the diseases. About 2 million new cases of leishmaniasis are reported each year. Sleeping sickness affects an estimated 50,000–500,000 people, mainly in rural sub-Saharan Africa.

## Nanotech to the Rescue?

A new study by the University of Toronto Joint Centre for Bioethics shows just how useful new nanotechnologies could be in helping developing countries overcome urgent problems such as extreme poverty, hunger, environmental degradation, and diseases such as malaria and HIV/AIDS. The study, published in the April 2005 *PLoS Medicine*, ranks nanotechnology applications by their potential contribution to development and meeting the eight UN Millennium Development Goals. The top 10 applications were deemed to be energy storage, production, and conversion; agricultural productivity enhancement; water treatment and remediation; disease diagnosis and screening; drug delivery systems; food processing and storage; air pollution and remediation; construction; health monitoring; and vector and pest detection and control. The study also noted that nanotechnology research and development initiatives have been launched in several developing countries.

## Africa Afire

By 2030, smoke from wood-fueled cooking fires will cause about 10 million premature deaths among African women and children, and by 2050, such fires will release 7 billion tons of carbon into the environment, according to a study published 1 April 2005 in *Science*. Sub-Saharan Africans consumed nearly 470 million tons of wood (in the form of firewood and charcoal) in 2000. Moving to petroleum-based fuels such as kerosene and propane gas would prevent the most premature deaths, but a more feasible strategy would be to adopt more modern methods of producing cleaner-burning charcoal. Such a shift could prevent 1–2.8 million premature deaths.

## Figures and Tables

**Figure:**
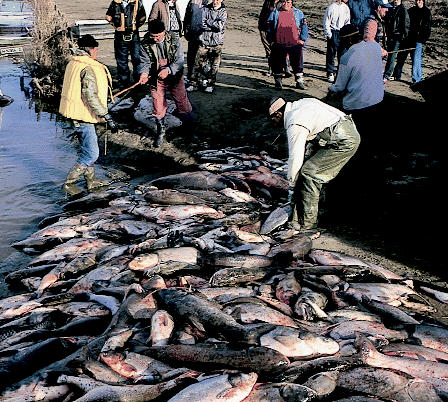


**Figure:**
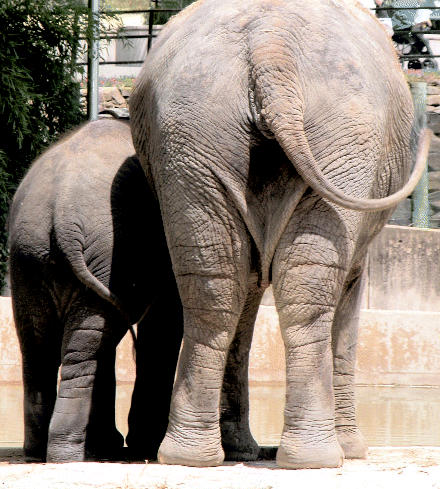


**Figure:**
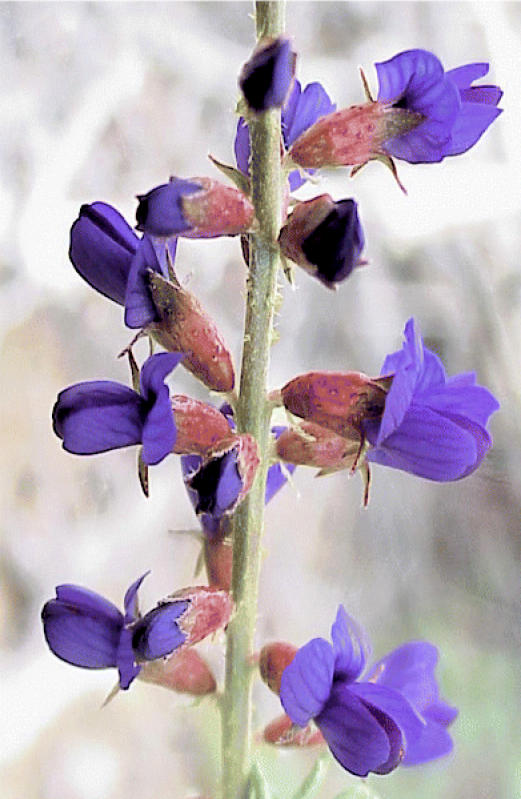


**Figure:**